# Incidence of Adverse Drug Reactions Among HIV Patients on Antiretroviral Drugs in Ethiopia: A Systematic Review and Meta-Analysis

**DOI:** 10.1155/arat/8820274

**Published:** 2025-03-17

**Authors:** Simachew Getaneh Endalamew, Solomon Keflie Assefa, Milkiyas Solomon Getachew, Fetlework Gubena Arage, Dejen Kahsay Asgedom, Bewuketu Terefe, Destaw Fetene Teshome

**Affiliations:** ^1^Department of Veterinary Epidemiology and Public Health, School of Veterinary Medicine, Bahir Dar University, P.O. Box 5501, Bahir Dar, Ethiopia; ^2^Department of Biostatistics and Epidemiology, Institute of Public Health, College of Medicine and Health Science, University of Gondar, Gondar, Ethiopia; ^3^Department of Public Health, College of Medicine and Health Science, Samara University, Samara, Ethiopia; ^4^Department of Community Health Nursing, School of Nursing, College of Medicine and Health Science, University of Gondar, Gondar, Ethiopia

**Keywords:** adverse drug reaction, antiretroviral therapy, HIV, incidence, meta-analysis, systematic review

## Abstract

**Introduction:** In recent decades, AIDS–related illnesses have declined due to the widespread use of highly active antiretroviral therapy (HAART). Despite the numerous benefits that HAART provides, it causes significant challenges for users in the form of adverse drug reactions (ADRs). Therefore, this systematic review and meta-analysis was conducted to explore the pooled incidence of antiretroviral therapy–related adverse drugs in Ethiopia.

**Methods:** A systematic review and meta-analysis was conducted on cohort studies conducted among HIV patients living in Ethiopia. The study was reported using the Preferred Reporting Items for Systematic Reviews and Meta-Analyses (PRISMA). A random-effects meta-analysis was used to determine the pooled incidence of ADR. Heterogeneity was assessed, and the source of variation was analyzed using subgroup and sensitivity analyses. Funnel plots and Egger's regression tests were used to investigate publication bias.

**Results:** Based on the random effects model from 10 extracted studies, the pooled incidence rate of adverse effects of ART drugs was 5.09 (95% CI: 3.86–6.71) per 100 person-years, with significant heterogeneity (*I*^2^ = 96.4%, *p* < 0.0001). It was also observed to be higher in subgroups from multicenter study areas, studies employing both prospective and retrospective designs, and among children.

**Discussion:** The findings of this systematic review and meta-analysis revealed disparities in ADR incidence rates. In addition, the findings of this review showed that the occurrence of ART–related ADR in people living with HIV is common in the healthcare system.

**Conclusion:** This systematic review and meta-analysis highlighted the significant incidence of adverse effects among individuals diagnosed with HIV in ART clinics in Ethiopia. A comprehensive strategy and coordinated collaboration among health planners, policymakers, and the community are essential to address this issue and integrate pharmacovigilance into service provision.

## 1. Introduction

The typical management strategy for individuals infected with human immunodeficiency virus (HIV) consists of administering combination therapies, which typically comprise more than one active ingredient of the antiretroviral agent [1]. The use of antiretroviral agents is accomplished through the combination of nucleoside reverse–transcriptase inhibitors, such as stavudine and zidovudine, with nonnucleoside reverse–transcriptase inhibitors such as nevirapine, efavirenz, and lamivudine. These regimens have resulted in substantial improvements in the morbidity and mortality rates for individuals infected with HIV [2]. Highly active antiretroviral therapy (HAART) has also enhanced the quality of life and treatment outcomes for individuals with HIV infection [3]. In recent decades, AIDS–related illnesses have declined due to the widespread use of HAART [4]. Despite the numerous benefits that HAART provides, it also causes significant challenges for users in the form of adverse drug reactions (ADRs [2]).

According to the World Health Organization (WHO) definition, an ADR refers to a detrimental occurrence or undesirable response associated with the utilization of a medicinal product [5]. Antiretroviral drugs can cause a wide range of toxicities, from potentially fatal side effects to low-grade intolerances that may go away on their own [6]. Antiretroviral drugs, like many medications taken over extended periods, are known to cause harmful effects and unwanted reactions. Commonly reported adverse reactions to antiretroviral medications encompass diarrhea, rash, neuropathy, hepatotoxicity, lactic acidosis, elevated liver enzymes, dyslipidemia, and insulin resistance [7–11].

The incidence of ADRs in patients receiving antiretroviral therapy (ART) differs across various geographic regions. Pieces of the literature revealed that the incidence proportion of ADR in people living with HIV ranges from 11% to 53% [12–17]. There is also a great variation in incidence rate reports across countries. According to a study by Shet et al. [18], the incidence density of ART–induced ADR in HIV patients in India was 52 and 15 per 100 person-years for all reactions and severe reactions, respectively. Prosperi et al. [19], from Italy, had reported an incidence density of 0.16 person-years with a 95% confidence interval (CI) of 0.14–0.18. Abah et al. [20], from Nigeria, has also reported an incidence rate of 2.83 with a 95% CI of 2.69–2.98 per 100 person-years. A study by Sherfa et al. [21] in Southern Ethiopia reported a 6.36 per 100 person-year incidence rate of ART–induced ADR in HIV patients.

Studies in Sub-Saharan Africa (SSA) have shown an increasing trend in the incidence of ADRs among individuals who are using ART for HIV treatment [22–25]. This geographic variation in ADR incidence among ART users can be attributed to differences in healthcare infrastructure and treatment practices. Resource-limited regions, such as SSA, often lack standardized laboratory monitoring systems, leading to delayed ADR detection and underreporting [26]. In addition, a higher burden of opportunistic infections and comorbidities, such as tuberculosis and malnutrition in this region, further exacerbate the risk of ADR [27]. Socioeconomic disparities and poor nutrition impair drug tolerance and metabolism, collectively elucidating the observed geographical disparities [28].

Several factors are associated with ART–related ADR occurrence, including age, gender, the timing of ARVs initiation, type of drug, drug–drug interactions, the presence of other diseases, decreased CD4 cell count, late WHO clinical stages, duration on treatment, disease biomarkers such as CD4 count and viral load, and body mass index (BMI) of the patients [29–31]. Moreover, concurrent treatment administration and polypharmacy may contribute to the development of ADR [32–36].

Even with the introduction of new generations of antiretroviral drugs to the market, managing patients with HIV remains difficult due to patient considerations such as pre-existing medical conditions, especially when it comes to reducing the risk of drug interactions [37]. ART–related ADRs are a significant contributor to medication switching, nonadherence, cessation, and unfavorable virologic outcomes [38–40]. Moreover, ART noncompliance and irregularity might result in drug resistance and even death [41]. A thorough monitoring strategy for ADRs connected to ART in HIV–positive individuals is urgently needed, given the increased frequency of ADRs in the Sub-Saharan region [42].

Following the UNAIDS 2020 target, which aimed to have 90% of individuals living with HIV aware of their status, 90% of individuals diagnosed with HIV receiving sustained ART, and 90% of those receiving ART achieve viral suppression ([43]). Several countries have implemented the WHO's “test and treat” policy. This policy recommends increasing the number of individuals with HIV receiving ART [44]. In addition, it advocates that ART should be immediately started in all individuals diagnosed with HIV regardless of age or CD4 cell count. Although this foundation was expected to shift the course of the epidemic, it faced challenges stemming from the increasing risks associated with different factors, which raise the likelihood of ADRs in people living with HIV [45]. Therefore, it is essential to conduct an empirical literature review starting from the introduction of the new paradigm WHO envisions, determining the incidence of ART–related ADRs and their implications for healthcare systems.

The current body of literature reveals a paucity of robust studies providing substantive insights into ART–related ADRs in Ethiopia, particularly in the form of systematic reviews and meta-analyses. Consequently, this systematic review and meta-analysis sought to aggregate the incidence rates of ART–related ADRs in Ethiopia. This analysis yields critical information regarding ADRs within the context of ART care and treatment, which may inform policymakers and program developers. This study contributes to the fundamental knowledge required to formulate strategies aimed at mitigating the risk of ADRs in Ethiopia.

## 2. Methods and Analysis

### 2.1. Development of the Review Method

The study was structured using the condition, context, and population (CoCoPop) framework [46]. The methods of this systematic review and meta-analysis protocol were developed based on the Preferred Reporting Items for Systematic Review and Meta-Analysis (PRISMA) Protocols 2020 statement [47]. The results of the review were also reported according to the PRISMA guidelines. The protocol was registered in an international prospective register of systematic reviews (PROSPERO) on February 21, 2024, with the registration number: CRD42024527240 focused on individuals diagnosed with HIV who attended ART clinics. The primary outcome of interest was the incidence of adverse effects of ART drugs. No specific interventions or comparator groups were included in this review.

### 2.2. Inclusion Criteria and Exclusion Criteria

All retrospective and prospective cohort studies conducted in Ethiopia were eligible for inclusion in this study. To ensure the incorporation of current and pertinent evidence on ADRs, only studies conducted after 2015 were included. Different study formats that reported the incidence rate of ADRs related to HIV were considered, including peer-reviewed journal articles, master's theses, and dissertations published in English.

Articles were excluded for one of the following reasons: (1) did not measure the outcome of interest; (2) they were investigating medication errors, medication overdose (intentional or accidental), and drug abuse; or (3) they were not related to ADR in ART specifically or are related to ART ADR but not to Ethiopian regions; and (4) the study sample size was below 100, to enhance the precision of the study.

In addition, limitations were implemented to include studies conducted after 2015 following the implementation of the WHO's “test and treat” policy. Studies published before 2015 were excluded to ensure consistency with the “test and treat” policy, which was implemented around this period and significantly altered diagnostic and treatment protocols. Restricting the analysis to more recent studies ensures that the findings reflect the near-current healthcare system and provide actionable insights relevant to ongoing public health interventions.

### 2.3. Searching Strategy

The academic and citation databases including Google Scholar (https://scholar.google.com), Embase (https://www.embase.com), PubMed (https://pubmed.ncbi.nlm.nih.gov), Web of Science (https://www.thomsonreuters.com), and Scopus (https://www.scopus.com/sources) were used for this systematic review and meta-analysis data source. The search was conducted on February 26, 2024. Records retrieved through the search were imported into the EndNote software, where duplicates were removed electronically and manually.

The search strategy followed the CoCoPop approach to identify relevant studies using search terms supplemented with added keywords and MeSH terms. The plan included terms related to the population (e.g., “HIV patients”), condition (e.g., “drug-related effects side” and “adverse reactions”), and context (e.g., “ART clinic” “highly active antiretroviral therapy”). Boolean operators such as “AND” and “OR” were applied to effectively combine terms. The search strategy, initially developed for PubMed, was adapted for other databases and included the following framework: (((((((((((incidence rate) OR (incidence)) AND (“adverse reactions”)) OR (“drug-related effects side”)) OR (“highly active antiretroviral therapy”)) OR (“HAART”))) OR ((“combination antiretroviral therapy”))) AND (HIV)) OR (human immunodeficiency virus)) OR (acquired immune deficiency syndrome virus)) OR (human T lymphotropic virus Type III) AND (Ethiopia [MeSH] OR Ethiopia).

### 2.4. Assessment of Methodological Quality

The quality of the studies was evaluated utilizing the Newcastle–Ottawa scale, a quality assessment tool that was modified to accommodate single-arm cohort studies [48, 49]. A quality score, modified from the Newcastle–Ottawa scale, was employed to evaluate the methodological rigor of the research design, sample representativeness, exposure ascertainment (demonstrating that the outcome of interest was not present at study initiation), nonexposed cohort selection, outcome determination reliability, follow-up duration, and cohort follow-up adequacy. The Newcastle–Ottawa scale does not prescribe a definitive threshold between high-, moderate-, and low-quality studies [50]. Therefore, a relative comparison of these studies was conducted.

Two reviewers (S.K.A. and S.G.E.) independently assessed the quality of the included studies. To address any discrepancies between the reviewers, discussions were held, and input was sought from other members of the review team, including F.G.A., D.K.A., M.S.G., D.F.T., and B.T. Score disagreements were resolved by consensus, and a final agreed-upon rating was assigned to each study.

### 2.5. Extraction of Data From Eligible Papers

The research findings from the searches were screened to ensure relevance, with only those that met the eligibility criteria selected for inclusion. Data extraction was performed using the standardized data extraction form, with Group 1 (S.K.A. and S.G.E.) and Group 2 (B.T., F.G.A., M.S.G., and D.K.A.) independently conducting the extraction. Any discrepancies in the extracted data between the two groups were resolved through discussion. The standardized data extraction form included information such as the first author's name, publication year, study region, study design, sample size, number of new adverse drug effect cases, total person-year observation, and incidence rate per 100 person-year observation were extracted for each included study. The data were summarized in a Microsoft Excel (Version 16.54) spreadsheet.

### 2.6. Operational Definitions

The dependent variable was the incidence of ART drug's adverse effect reactions among HIV–positive patients. An incident adverse drug, or event of interest in this study, is defined as the occurrence of at least one of the following undesirable effects registered in the patient's follow-up card (nausea, diarrhea, fatigue, headache, numbness/tingling/pain, skin rash, anemia, abdominal pain, jaundice, fat change, dizzy, anxiety, nightmare, and depression). For this study, only the first event that occurred following the date of the first prescription of antiretroviral drugs was under investigation.

### 2.7. Data Synthesis and Statistical Analysis

The incidence rates were expressed per hundred person-years. To standardize incidence rates across studies that reported different time units, we converted all rates to a common unit of per 100 person-years. Incidence rates reported per 100 person-months were multiplied by 12, and those reported per 100 person-days were multiplied by 365, using the following formulas:

(1) $$\begin{array}{c} \text{incidence per }100\text{ person}-\text{years}=\text{incidence per }100\text{ person}-\text{months}\times 12,\\ \text{incidence per }100\text{ person}-\text{years}=\text{incidence per }100\text{ person}-\text{days}\times 365. \end{array}$$

For studies that did not report 95% CIs, these were calculated using the following standard formula [51]:

(2) $$\begin{array}{c} \text{LCI}=e^{\ln\left(\text{IR}\right)-1.96\sqrt{\left(1/E\right)}}\text{ while},\text{ UCI}=e^{\ln\left(IR\right)+1.96\sqrt{\left(1/E\right)}}, \end{array}$$

where IR is the  incidence rate, LCI is the lower CI, UCI is the upper CI, and *E* is the number of events who developed ADR.

All quantitative analyses were performed by the “Meta” package (Version 8.0-1) from RStudio software (Version 2024.12.0-467). The decision between fixed and random effects models was first guided by an assessment of between-study heterogeneity. Given the substantial heterogeneity observed (*I*^2^ = 96.4%), a random effects model was selected. Among the random effects models, the most suitable transformation method was chosen by assessing the normality of the data using five approaches including original rate without transformation (PR), arcsine conversion (PAS), double-arcsine transformation (PFT), logarithmic conversion (PLN), and logit transformation (PLOGIT). The final model was selected based on the Shapiro–Wilk normality test results, where a W-value close to 1 and a *p* value > 0.05 indicated a closer fit to the Gaussian distribution. Based on this assessment, PLOGIT transformation was chosen, as it yielded a W-value of 0.95858 and a *p* value of 0.7539, indicating that the transformed data closely followed a Gaussian distribution.

A random-effects meta-analysis was conducted using inverse variance weighting, with heterogeneity estimated using the DerSimonian–Laird method [52]. To enable pooling, the log incidence rate of ADRs and corresponding standard error were calculated for each study using the inverse variance method [51, 53]. To adjust the CI of the pooled estimate, the Hartung–Knapp–Sidik–Jonkman method was applied [54, 55]. Between-study heterogeneity variance and its uncertainty were quantified using CIs for *τ*^2^ (tau^2^) and *τ* (tau) derived from the Jackson method [56]. Finally, a forest plot was used to visually examine the distribution of incidence rates and their 95% CIs across studies. Variability among the included studies, stemming from differences in study quality, sample size, methodology, and outcome measures, was evaluated using the Cochrane Q-test [57], *I*^2^ statistics [58], and prediction interval [59] to assess heterogeneity in the meta-analysis. Subgroup analysis was conducted based on study regions, study participants (adult or children), publication year, and study design to minimize the variance of estimated points between primary studies. Sensitivity analysis was also performed to assess the influence of individual studies on the pooled estimates. Univariable meta-regression was conducted using mean age, the year of publication, sample size, and study regions, using the random effects model. Publication bias was assessed graphically and with Egger's statistical test. A statistically insignificant Egger's test (*p* value > 0.05) indicates the absence of a small study effect.

## 3. Results

### 3.1. Study Selection and Identification

**Figure 1:**
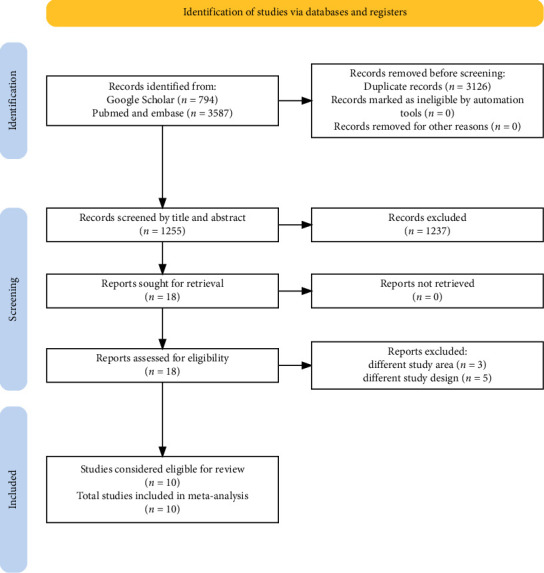
PRISMA flow diagram for study selection (identification, screening, eligibility assessment, and inclusion of studies) in the systematic review and meta-analysis.

Of the 4381 studies initially screened, 3126 were removed due to duplication. A further 1235 studies were excluded based on their titles and abstracts, as they were considered irrelevant to the review. In addition, eight studies were excluded for reasons such as not reporting the correct primary outcome result, different study areas, and inadequate sample size. Finally, 10 studies were considered suitable for inclusion in the qualitative and quantitative syntheses, as depicted in the PRISMA flow diagram (Figure 1).

### 3.2. Description of the Included Studies

This study included data from 10 studies published between 2015 and 2024 in Ethiopia, encompassing a total of 8333 patients on ART, of whom 1603 experienced ADRs related to ART. Southern Nations, Nationalities, and Peoples' Region (SNNPR) and Amhara have contributed most studies of ADR due to ART in Ethiopia. Most of the studies were retrospective cohort studies with follow-up periods ranging from 1 to 4 years. The sample sizes varied from 376 to 3921 individuals. The incidence rate of ART adverse effects ranged from 3.04 to 9.00 per 100 person-years in the study area (Table 1).

### 3.3. The Pooled Incidence Rate of ART–Induced Adverse Effects

**Figure 2:**
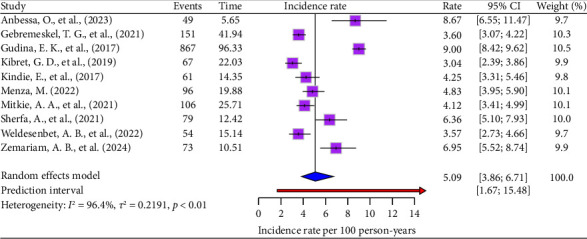
Forest plots of the incidence rate of ART drug's adverse effects per 100 person-years.

This meta-analysis identified heterogeneity between the included studies (*I*^2^ = 96.4% [95% CI: 94.8–97.5], *p* < 0.001 and *τ*^2^ = 0.22 [95% CI: 0.07–0.63], *p* value = 0.01). As a result, we used a random effects model to estimate the pooled incidence rate of ART drug's adverse effects. Based on the random effects model, the pooled incidence rate of ART drug's adverse effects per 100 person-year observations among HIV–infected persons was 5.09 (95% CI: 3.86–6.71) (Figure 2).

### 3.4. Handling Heterogeneity

The between-study heterogeneity revealed a significant result (*Q* = 248.14, df = 9, *p* < 0.0001), indicating substantial variability in effect sizes across the included studies and the variance was estimated at *τ*^2^ = 0.22 (95% CI: 0.07–0.63), with an *I*^2^ value of 96.4% (95% CI: 94.8–97.5).

Several methodologies, including sensitivity analysis, subgroup analysis, and meta-regression analysis, were employed to address the substantial heterogeneity observed in the pooled estimate derived from the random effects model. These methods help to explore potential sources of heterogeneity and provide insights into the robustness of the findings.

#### 3.4.1. Subgroup Analyses of ART Drug's Adverse Effect

Subgroup analyses were conducted to explore ART drug's adverse effect incidence rates among HIV–infected individuals. The analyses showed that the study area or region, study design, and participants have significant variations in the between-study heterogeneity analysis. However, the category of publication year subgroup difference was insignificant (Table 2).

Among the regional subdivisions, the multicenter study area reported the highest incidence rate of ART–induced ADRs among people living with HIV/AIDS, at 9 per 100 person-years (95% CI: 8.42–9.62). This was followed by the Harari region, with an incidence rate of 8.67 (95% CI: 6.55–11.47). In contrast, the Oromia region reported the lowest incidence rate, at 3.57 per 100 person-years (95% CI: 2.73–4.66), though this finding was based on a single study from the region.

**Figure 3:**
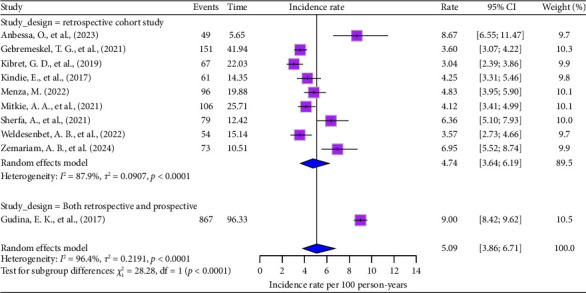
Subgroup analysis based on study design.

Regarding study design, retrospective studies revealed the lowest ADR incidence rate of 4.74 (95% CI: 3.64–6.19) per 100 person-years, with significant heterogeneity (*I*^2^ = 88%, *p* < 0.0001) (Figure 3).

#### 3.4.2. Sensitivity and Influence Analyses

**Figure 4:**
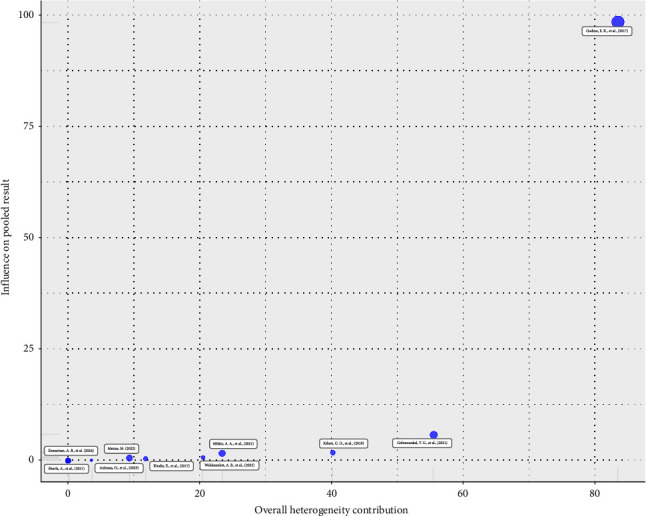
Baujat diagnostic plots of ADR among ART users in Ethiopia.

Baujat diagnostic plots were used to detect studies that overly contribute to the heterogeneity in a meta-analysis. The plot shows the contribution of each study to the overall heterogeneity (as measured by Cochran's *Q*) on the horizontal axis and its influence on the pooled effect size on the vertical axis [69]. Accordingly, a study by Gudina et al. [63] contributed heavily to the overall heterogeneity in our meta-analysis (Figure 4).

**Figure 5:**
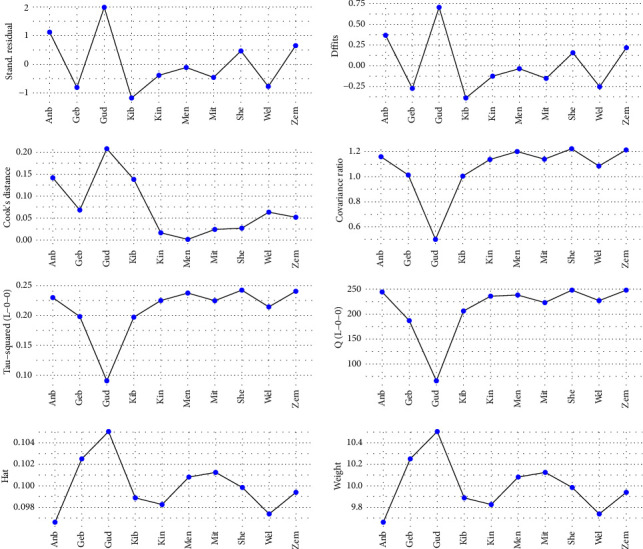
Influence diagnostic plots of the included studies.

Influence diagnostics were also performed graphically using various diagnostic tests for each included study. Higher values of difference in fits (DFFITS), standardized residuals, and Cook's distance were found in a study by Gudina et al. [63], indicating that this may be an influential case because its impact on the average effect is larger. The covariance ratio value was also below one for this study, indicating that removing this study could result in a more precise estimate of the pooled effect size. In addition, leave-one-out *τ*^2^ and *Q* values that estimate heterogeneity as measured by *τ*^2^ and Cochran's *Q* are also lower if this study is removed, resulting in lower heterogeneity. In the last row, we saw that the study weight and hat value for this study were extremely larger indicating that the study is an influential case and may negatively affect the robustness of our pooled result (Figure 5).

Finally, a leave-one-out analysis was conducted to evaluate the influence of a single study on the overall effect size estimate. In this analysis, the corresponding study was excluded, and a meta-analysis was performed on the remaining (*n* − 1) studies. If the CI of the study did not encompass the overall effect size estimate, it was deemed that the study had a substantial impact on it [70]. In this study, the overall effect size estimate was 5.09, which fell within the CIs of all the studies. The sensitivity test showed that the combined pooled incidence rate was not significantly affected by the omission of any of the studies. Thus, the meta-analysis results encompassing all included studies in this study were reliable.

**Figure 6:**
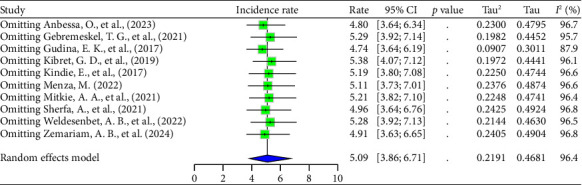
Sensitivity analysis of the ADR incidence rate among HIV–infected persons in Ethiopia.

However, the study by Gudina et al. [63] was identified as an influential outlier among the included studies. Although omitting this study did not significantly impact the combined pooled incidence rate, as demonstrated by the sensitivity analysis, it substantially reduced the between-study heterogeneity (*τ*^2^ = 0.0907 [95% CI: 0.0382–0.4038] and *I*^2^ = 87.9% [95% CI: 79.3%–93.0%]) (Figure 6).

#### 3.4.3. Meta-Regression

Univariable meta-regression was conducted using the mean age of participants, year of publication, and sample size as a continuous variable and study regions, study participants, and study design as categorical variables using the mixed effects model. These variables were subjected to assessment to see a linear relationship with the dependent variable, which is the effect size. Those variables with a *p* value < 0.1 were declared to be significant at the univariable analysis for multivariable meta-regression (Table 3).

Based on this, only the study design was found to be significant at *p* values < 0.1. The meta-regression model by study design explains 58.61% of the heterogeneity. The model estimated the regression weight for retrospective cohort studies to be −0.64. This means that retrospective cohort studies are associated with lower effect sizes, without controlling for other variables compared to both retrospective and prospective cohort studies.

### 3.5. Prediction Interval

A prediction interval provides information on the anticipated variation in outcomes of a new study if it were selected randomly from the same set of studies utilized in the present analysis. It reflects the uncertainty we expect in the summary effect if a new study is included in the meta-analysis [71, 72]. As per the findings of this systematic review and meta-analysis, the prediction interval ranged from *g* = 1.63 to 15.82 per 100 person-years. Therefore, future studies on ADRs among ART users are likely to produce results within these established ranges.

### 3.6. Publication Bias

**Figure 7:**
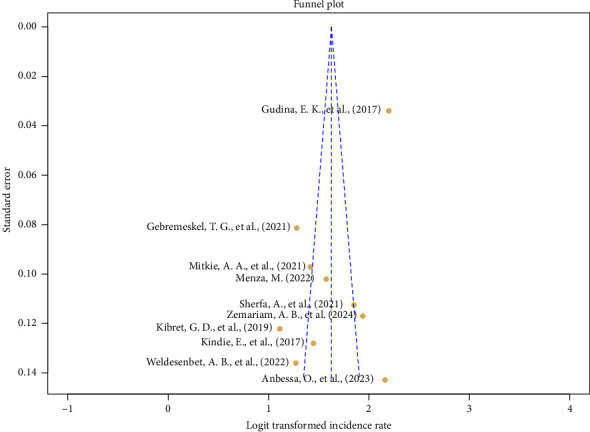
Funnel plot of the pooled incidence rate of ART drug's adverse effect reaction.

We assessed publication bias using both graphical and statistical tests [73]. The funnel plot displayed an asymmetrical pattern. In addition, there were notable outliers, such as the studies by Anbessa et al. [60], which had high standard errors, and Gudina et al. [63] which had the greatest precision and extremely high effect size (Figure 7).

Publication bias can lead to asymmetrical funnel plots and statistically significant Egger's test results. However, alternative factors may produce similar patterns [74]. One such factor is between-study heterogeneity. Funnel plots typically assume that the dispersion of effect sizes is attributable to sampling errors alone, disregarding the possibility that studies might estimate different underlying true effects.

Since the funnel plot provides a visual assessment, it is also beneficial to assess asymmetry quantitatively using Egger's regression test [75]. The Egger's test was conducted to evaluate the presence of publication bias. The test yielded a *p* value of 0.0142 and a bias estimate of −7.37 when incorporating all studies, including an outlier study by Gudina et al. [63]. However, upon exclusion of this influential outlier, the Egger's test produced nonsignificant results (0.2892), indicating that the observed test statistics and funnel plot asymmetry were more likely attributable to between-study heterogeneity rather than small-study effects.

## 4. Discussion

This review offers a current and thorough systematic evaluation of the incidence of ADRs related to ART in Ethiopia. Research studies encompassing the incidence of ADRs in HIV–infected patients across different regions were incorporated to determine the pooled incidence rate of ADRs in HIV patients. Based on this goal, 10 studies published between 2015 and 2024 in scientific and reputable journals and unpublished articles were included for systematic review and meta-analysis study. The findings of this systematic review and meta-analysis study revealed disparities in the incidence rate of ADR. In addition, the findings of this review showed that the occurrence of ART–related ADR in people living with HIV is common in the healthcare system.

The study's lowest rate of ADR was 3.04 per 100 person-years (95% CI: 2.39–3.86) in Amhara, and the highest rate was 9.00 per 100 person-years with (95% CI: 8.42–9.62) from multicenter studies. The findings of this systematic review and meta-analysis study revealed substantial heterogeneity among the included studies. In our heterogeneity analysis using different diagnostics, the study by Gudina et al. [63] was identified as an influential outlier. Sensitivity analysis demonstrated that excluding this study did not significantly alter the pooled incidence rate, confirming the robustness of our findings. However, its removal substantially reduced between-study heterogeneity (*τ*^2^ = 0.0907; 95% CI: 0.0382–0.4038 and *I*^2^ = 87.9%; 95% CI: 79.3%–93.0%), suggesting that this study contributes notably to the observed variability. The heterogeneity may arise from differences in study duration, ART regimens used, or population characteristics, which are common in meta-analyses of this nature. Despite the remaining heterogeneity, the stability of the pooled results strengthens confidence in our conclusions.

The findings of this study showed that the pooled incidence rate of ADR among HIV–positive patients was 5.09 per 100 person-years (95% CI: 3.86–6.71). This result is much less than earlier studies conducted in South Africa [76], which reported 36.7 per 100 person-years of incidence, and a study conducted in India [18], which reported a rate of 52 per 100 person-years for all forms of ADR reactions. The heterogeneity in patient outcomes across various ART studies can be attributed to disparities in monitoring duration, treatment regimens, clinical stage at the initiation of therapy, socioeconomic disparities between developed and developing nations, and variations in the severity of adverse reaction types reported [21, 77].

The findings of the current study are also greater than the study in Italy [19] and Nigeria [20], which had reported incidence rates of 0.16 (95% CI: 0.14–0.18) and 2.83 (95% CI: 2.69–2.98) per 100 person-years, respectively. The reasons for these differences could be attributed to the fact that this study had included both adult and child patients, the varying socioeconomic status of the study subjects, variations in health system policies and their implementation, and the differences in the healthcare systems available for monitoring ADRs across different regions [21]. This is supported by evidence showing that roughly 43% of ADRs are preventable, which implies a complex interplay among the healthcare system's various actors responsible for ensuring the safe delivery of medications [78].

The study emphasized that ADRs' incidence is a big challenge and had significant consequences on the healthcare system. Cases of severe and potentially fatal ADRs have resulted in changes to medication regimens and situations where patients have been temporarily discontinued from their medications due to limited treatment options [79].

Severe ADRs can result in a range of clinical manifestations, from hospitalization to life-threatening reactions, necessitating comprehensive strategies to mitigate their occurrence. This issue is particularly critical in populations with complex comorbidities, polypharmacy, or those undergoing ART, wherein drug interactions and adverse effects are prevalent. The consequences of ADRs are not limited to clinical outcomes; they also have significant financial and resource implications, as managing these reactions often necessitates additional healthcare resources including monitoring, testing, and extended treatment durations.

### 4.1. Strengths and Limitations of the Review

The strength of this systematic review lies in its extensive step-by-step adherence to procedures for acquiring quality evidence. The review also followed the PRISMA reporting guidelines for systematic reviews and used available filters to ensure that no information was left out. Multiple databases were searched, and a rigorous process was followed to select the studies. Moreover, the methodological quality of each included study was assessed using universally approved tools for incidence studies.

This study has limitations, including differences in demographic and socioeconomic factors, study duration, and ART regimens used which may contribute to the unexplained variability between these studies. Although subgroup analysis was used to explore sources of this variability, no consistent sources were identified across all groups, despite the ability to calculate pooled estimates.

The significant heterogeneity in this meta-analysis could be influenced by factors that were not fully captured by regional or participant-based subgroup analyses. One potential source of variability could be the difference in ART regimens and dosing schedules across studies. Older ART drugs are often associated with higher toxicity. In contrast, newer formulations aim to reduce adverse effects, leading to potentially different ADR rates depending on the regimen used in each study [80]. Access to healthcare and the rigor of monitoring protocols also vary and could affect ADR reporting. In regions or facilities with limited resources, less severe ADRs may go undetected or unreported, whereas studies with more comprehensive follow-up practices may record higher ADR rates. Consequently, this disparity in healthcare quality and provider training can contribute to inconsistent ADR findings across studies [81].

Demographic and socioeconomic factors such as nutritional status, comorbid conditions, and genetic predispositions might also influence the susceptibility of patients to ADRs. Malnutrition or concurrent illnesses, for example, can exacerbate side effects, and genetic differences in drug metabolism may result in different ADR profiles between populations. These factors often vary by region and are generally unaccounted for in meta-analyses, contributing to the heterogeneity of reported outcomes [82].

Differences in the study quality and data collection methods, particularly between retrospective and prospective studies, can further contribute to variability. Retrospective studies might rely on incomplete records, leading to underreporting, whereas prospective studies often have structured data collection, capturing a broader range of ADRs. These methodological discrepancies might create variability in the reported incidence rates. Another factor is potential selection and reporting bias. Studies with higher ADR incidence rates might be more likely to be published, whereas studies with lower rates remain unpublished, potentially inflating the heterogeneity between studies. Selective reporting of significant ADRs over minor ADRs can also skew the data, resulting in higher variability across studies.

Finally, patient adherence and behavior following ART regimens play a role in ADR rates. Inconsistent ART usage due to side effects or lifestyle factors can reduce ADR incidence simply due to less consistent drug exposure yet may also cause specific reactions if doses are resumed irregularly. Differences in adherence across studies can, therefore, contribute to varied ADR findings, impacting overall heterogeneity. Collectively, these factors suggest that the observed heterogeneity in ADR incidence rates is multifactorial and influenced by a combination of drug-related, healthcare, demographic, methodological, and behavioral elements, making it challenging to isolate specific causes in the meta-analysis framework.

Another limitation of this study is the restriction on English-language publications, which may have introduced language bias. This could have resulted in the exclusion of relevant studies published in other languages, potentially affecting the generalizability of our findings. Future research should include studies in multiple languages to provide a more comprehensive analysis.

## 5. Conclusions

This systematic review and meta-analysis highlighted a significant incidence of adverse effects among individuals diagnosed with HIV attending ART clinics in Ethiopia. The pooled incidence rate of ART drug's adverse effects was 5.09 per 100 person-year observations, with the highest incidence from multicenter studies and the lowest in the Amhara region. A comprehensive strategy and coordinated collaboration among health planners, policymakers, and the community are essential to address this issue and integrate pharmacovigilance into service provision.

The findings of this study underscore the critical importance of addressing the ongoing challenges of ADRs in clinical practice. A comprehensive, multifaceted approach is needed to reduce the incidence, minimize harm, and ensure the best possible outcomes for patients. Clinicians and policymakers must work together to develop and implement strategies that prioritize patient safety, enhance drug monitoring, and provide safe and effective alternatives for patients who experience ADRs. This collective effort will help alleviate the burden of ADRs on both the healthcare system and its patients.

To enhance the management of ADRs in clinical practice, it is crucial for clinicians to implement rigorous monitoring systems for early detection, particularly in high-risk populations, and to adopt personalized medicine approaches that take into account genetic factors and comorbidities in order to minimize ADR risks. Patient education should be prioritized to empower individuals in self-monitoring, while clinicians must be prepared with alternative therapies and clear guidelines for safely adjusting treatments when severe ADRs occur. In addition, integrating clinical decision support systems within electronic health records can assist healthcare providers by flagging potential drug interactions, tracking ADRs, and recommending safer treatment alternatives based on individual patient histories.

On the policy front, strengthening ADR management requires the establishment of comprehensive national ADR reporting systems to enable real-time tracking, improving access to affordable alternative medications for those affected by severe ADRs, and adopting standardized guidelines for ADR management alongside continuous training for healthcare providers. Regulatory agencies should enhance pharmacovigilance efforts, focusing on postmarket surveillance to ensure prompt access to ADR data, while governments should allocate sufficient resources to fund patient safety programs, drug safety research, and effective monitoring systems both at institutional and national levels.

## Figures and Tables

**Table 1:** Characteristics of the included studies and incidence of ART drug's adverse effect per 100 person-year in Ethiopia, 2024 (*n* = 10).

Author and publication year	Study region	Study design	Sample size	Events	Total person-year	⁣^∗^IRPY100
Anbessa et al. (2023) [60]	Harari	Retrospective cohort study	449	49	565	8.67
Zemariam et al. (2024) [61]	Amhara	Retrospective cohort study	439	73	1050.59	6.95
Gebremeskel et al. (2021) [62]	Tigray	Retrospective cohort study	452	151	4194.44	3.60
Gudina et al. (2017) [63]	Multicenter	Both retrospective and prospective	3921	867	9633.33	9.00
Kibret et al. (2019) [64]	Amhara	Retrospective cohort study	485	67	2202.7	3.04
Kindie et al. (2017) [65]	Amhara	Retrospective cohort study	602	61	1435	4.25
Menza (2022) [66]	SNNPR	Retrospective cohort study	376	96	1988	4.83
Mitkie et al. (2021) [67]	SNNPR	Retrospective cohort study	592	106	2571	4.12
Sherfa et al. (2021) [21]	SNNPR	Retrospective cohort study	456	79	1241.9	6.36
Weldesenbet et al. (2022) [68]	Oromia	Retrospective cohort study	561	54	1514.2	3.57

*Note:*⁣^∗^IRPY100 = incidence rate per 100 person-years.

**Table 2:** ART drug's adverse effect reaction incidence rate in HIV–positive people in Ethiopia: Subgroup meta-analysis and heterogeneity analysis.

Variables		Included studies	Incidence per 100 person-year with 95% confidence interval (CI)	Heterogeneity (*I*^2^)	*p* value
Region	Tigray	1	3.6 (CI: 3.07; 4.22)	—	< 0.0001
	Amhara	3	4.48 (1.59; 12.61)	91.7%	
	Oromia	1	3.57 (CI: 2.73; 4.66)	—	
	SNNPR	3	5.00 (2.91; 8.59)	76.6%	
	Harari	1	8.67 (CI: 6.55; 11.47)	—	
	Multicenter	1	9.00 (CI: 8.42; 9.62)	—	

Study design	Retrospective	9	4.74 (3.64; 6.19)	88%	< 0.0001
	Both	1	9.00 (8.42; 9.62)	—	

Study participants	Children	1	6.95 (5.52; 8.74)	—	0.0487
	Adults	9	4.91 (3.63; 6.65)	96.8%	

**Table 3:** Univariable meta-regression analysis results for the incidence of ADR in Ethiopia.

Variables		⁣^∗^*R*^2^	Coefficient (95% CI)
Mean age		0.00%	−0.01 (−0.04; 0.024)

Regions	Amhara	58.89%	Reference
	Tigray		−0.22 (−1.26; 0.82)
	Oromia		−0.23 (−1.32; 0.86)
	SNNPR		0.11 (−0.64; 0.86)
	Harari		0.66 (−0.43; 1.75)
	Multicenter		0.70 (−0.32; 1.71)

Study design	Both	58.61%	Reference
	Retrospective		−0.64 (1.45: 2.95)

Sample size		0.00%	0.0002 (−0.0001; 0.0004)

Publication year		5.96%	0.02 (−0.12; 0.15)

Study participants	Adults	0.00%	Reference
	Children		0.35 (−0.61; 1.31)

*Note:*⁣^∗^*R*^2^ = coefficient of determination.

## Data Availability

All datasets are included within the manuscript or as supporting files.

## Nomenclature

ADR: Adverse drug reaction
AIDS: Acquired immunodeficiency syndrome
ART: Antiretroviral therapy
CoCoPop: Condition, context, and population
HAART: Highly active antiretroviral therapy
PRISMA: Preferred Reporting Items for Systematic Review and Meta-Analysis
SNNPR: Southern Nations, Nationalities, and Peoples' Region
WHO: World Health Organization
